# Graphitic carbon nitride prepared from urea as a photocatalyst for visible-light carbon dioxide reduction with the aid of a mononuclear ruthenium(II) complex

**DOI:** 10.3762/bjoc.14.153

**Published:** 2018-07-17

**Authors:** Kazuhiko Maeda, Daehyeon An, Ryo Kuriki, Daling Lu, Osamu Ishitani

**Affiliations:** 1 Department of Chemistry, School of Science, Tokyo Institute of Technology, 2-12-1-NE-2 Ookayama, Meguro-ku, Tokyo 152-8550, Japan; 2 Japan Society for the Promotion of Science, Kojimachi Business Center Building, 5-3-1, Kojimachi, Chiyoda-ku, Tokyo 102-0083, Japan; 3 Suzukakedai Materials Analysis Division, Technical Department, Tokyo Institute of Technology, 4259 Nagatsuta-cho, Midori-ku, Yokohama 226-8503, Japan

**Keywords:** artificial photosynthesis, heterogeneous photocatalysis, hybrid material, metal complexes, solar fuels

## Abstract

Graphitic carbon nitride (g-C_3_N_4_) was synthesized by heating urea at different temperatures (773–923 K) in air, and was examined as a photocatalyst for CO_2_ reduction. With increasing synthesis temperature, the conversion of urea into g-C_3_N_4_ was facilitated, as confirmed by X-ray diffraction, FTIR spectroscopy and elemental analysis. The as-synthesized g-C_3_N_4_ samples, further modified with Ag nanoparticles, were capable of reducing CO_2_ into formate under visible light (λ > 400 nm) in the presence of triethanolamine as an electron donor, with the aid of a molecular Ru(II) cocatalyst (RuP). The CO_2_ reduction activity was improved by increasing the synthesis temperature of g-C_3_N_4_, with the maximum activity obtained at 873–923 K. This trend was also consistent with that observed in photocatalytic H_2_ evolution using Pt-loaded g-C_3_N_4_. The photocatalytic activities of RuP/g-C_3_N_4_ for CO_2_ reduction and H_2_ evolution were thus shown to be strongly associated with the generation of the crystallized g-C_3_N_4_ phase.

## Introduction

Carbon nitride is one of the oldest synthetic polymers [1], and has several allotropes. Among them, graphitic carbon nitride (g-C_3_N_4_) is the most stable form and is an emerging material as an organic semiconductor photocatalyst active for various kinds of reactions such as water splitting, CO_2_ reduction, and degradation of harmful organic compounds, because of its non-toxic, stable, and earth-abundant nature [2–7].

Our group has developed photocatalytic CO_2_ reduction systems using g-C_3_N_4_-based materials, in combination with functional metal complexes [8–16]. For example, mesoporous g-C_3_N_4_ (mpg-C_3_N_4_) modified with a mononuclear Ru(II) complex, such as *trans-*(Cl)-Ru{(PO_3_H_2_)_2_bpy(CO)_2_Cl_2_} (bpy: 2,2’-bipyridine), abbreviated as RuP, is capable of photocatalyzing CO_2_ reduction into formate with high selectivity under visible light irradiation, as confirmed by isotope tracer experiments with ^13^CO_2_ [8–12]. After the first report of a metal complex/C_3_N_4_ hybrid for CO_2_ reduction, several groups have presented similar reports using cobalt-based metal complexes as reduction cocatalysts [17–20].

In these systems, structural properties of g-C_3_N_4_ such as specific surface area and porosity have a strong impact on activity, because they strongly affect the efficiency of electron/hole utilization to the surface chemical reactions [21–22]. Apart from mpg-C_3_N_4_ that is usually prepared by a hard-template method with multistep procedures [9,23], g-C_3_N_4_ having a relatively higher surface area can be readily prepared by heating urea, which is an inexpensive and readily available precursor, in air [14,24]. In fact, the urea-derived g-C_3_N_4_ exhibited an enhanced activity for CO_2_ reduction compared to mpg-C_3_N_4_, when modified with Ag nanoparticles and a binuclear Ru(II) complex [14].

**Scheme 1:**
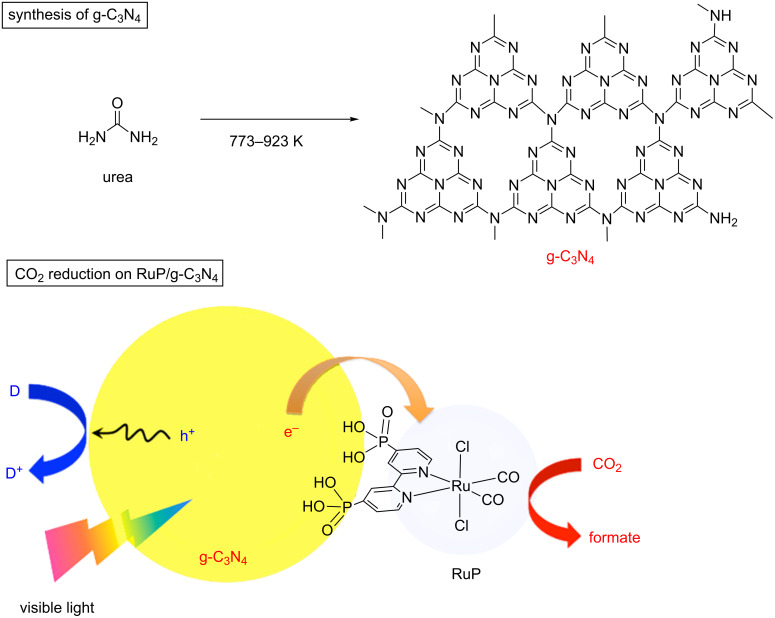
Synthesis of g-C_3_N_4_ by thermal heating of urea and application to photocatalytic CO_2_ reduction with a mononuclear Ru(II) complex (RuP).

Thermal heating of urea results in decomposition and formation of g-C_3_N_4_, whose physicochemical properties should be dependent on the heating temperature. In this work, we investigated photocatalytic activities of g-C_3_N_4_, which was synthesized by heating urea at different temperatures, for visible-light CO_2_ reduction with the aid of a mononuclear Ru(II) complex, RuP (see Scheme 1). As mentioned earlier, g-C_3_N_4_ has been studied as a visible-light-responsive photocatalyst mostly for H_2_ evolution from aqueous triethanolamine (TEOA) solution [2–3,5]. The present work also compares the activities for CO_2_ reduction with those for H_2_ evolution in order to obtain a better understanding on photocatalytic activities of g-C_3_N_4_ for different kinds of reactions.

## Results and Discussion

### Synthesis of g-C_3_N_4_ by thermal heating of urea at different temperatures

**Figure 1:**
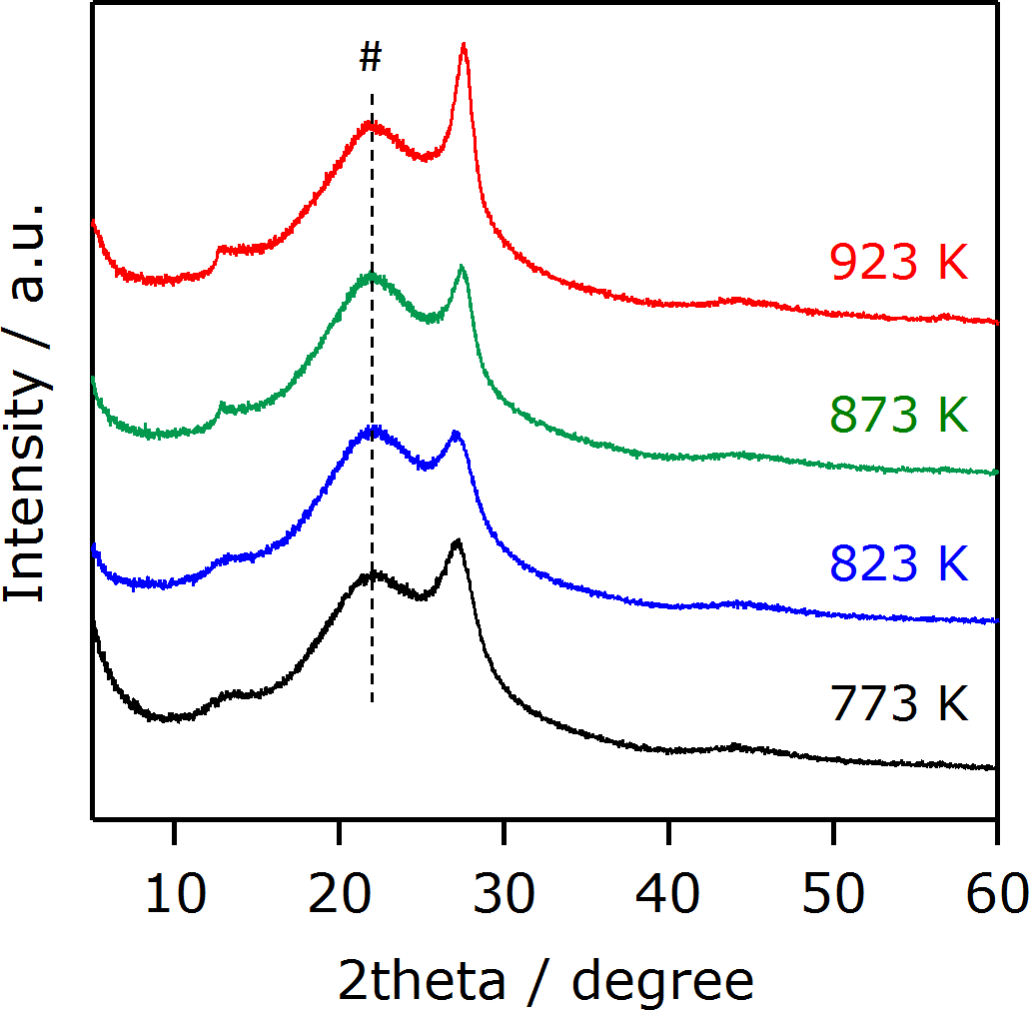
XRD patterns of g-C_3_N_4_ synthesized at different temperatures. A broad peak at around 22 degree, indicated by #, in XRD patterns originated from a glass folder for the measurement.

Figure 1 shows XRD patterns of g-C_3_N_4_ samples synthesized at different temperatures. Two peaks are observed at 2theta = 13 and 27.4**°**, which are assigned to an in-planar repeating motif and the stacking of the conjugated aromatic system, respectively [25]. This result confirms the successful synthesis of g-C_3_N_4_ at the present temperature range examined. With increasing temperature, the intensity of these peaks became stronger, indicating that the formation of g-C_3_N_4_ was facilitated at higher temperatures. It is, however, noted that the high-temperature heating also caused the loss of the product mass due to the decomposition of g-C_3_N_4_ itself [25].

**Figure 2:**
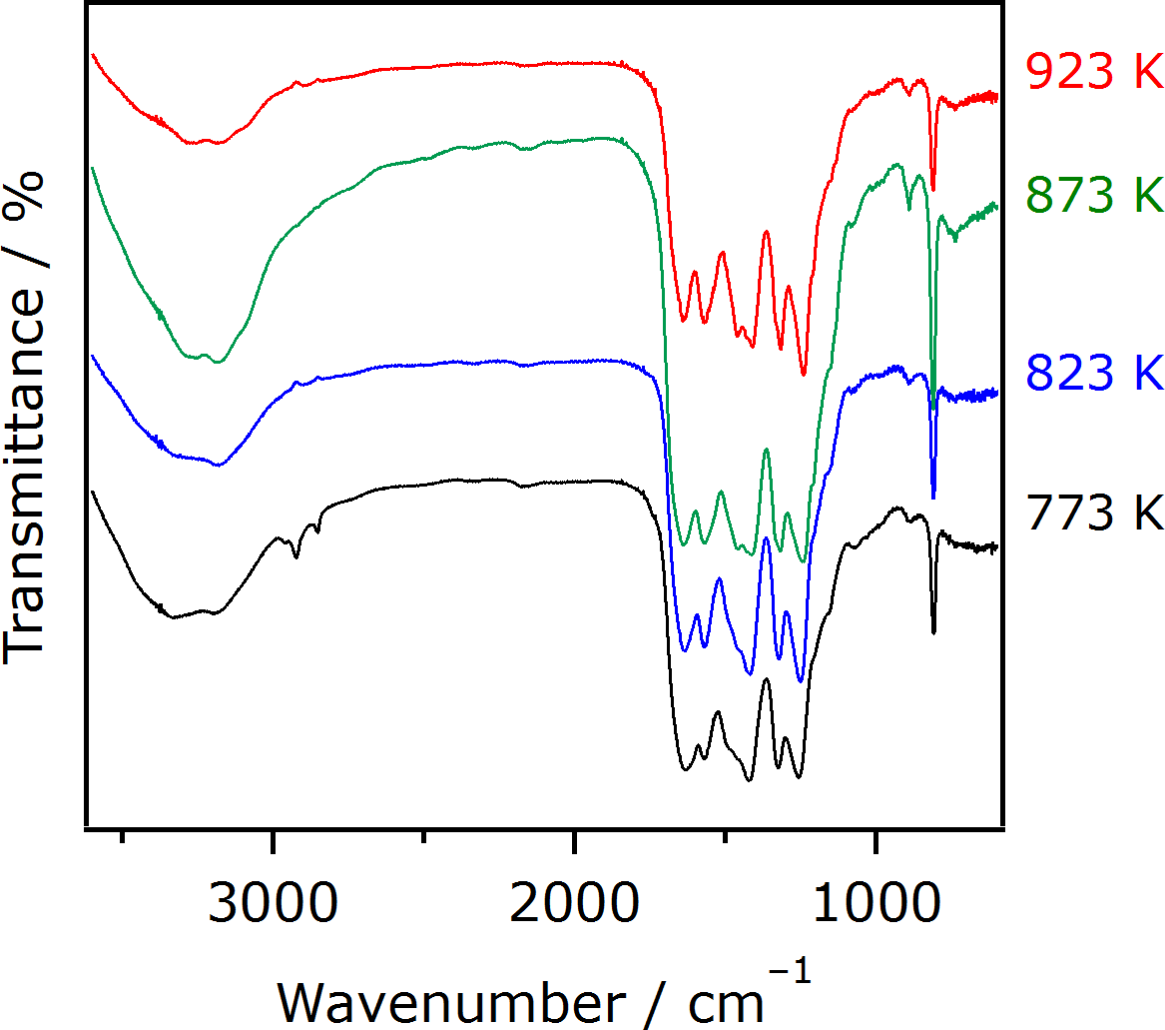
FTIR spectra of g-C_3_N_4_ synthesized at different temperatures. Each spectrum was acquired by a KBr method in N_2_ atmosphere.

FTIR spectra for the same set of the samples are shown in Figure 2. Characteristic peaks can be seen in the 1650–1200 cm^−1^ region. The peaks at 1322 and 1243 cm^−1^ are assigned to the stretching vibration of connected units of C–N(–C)–C (full condensation) or C–NH–C (partial condensation) [26–27]. The leftovers of 1641, 1569, 1462 and 1412 cm^−1^ are assigned to stretching vibration modes of heptazine-derived repeating units, and are sharper with increasing temperature. This further indicates the production of g-C_3_N_4_ at elevated temperatures, consistent with the XRD analysis (Figure 1). The 812 cm^−1^ peak is attributed to the out-of-plane bending vibration characteristic of heptazine rings.

The results of elemental analyses for the as-prepared g-C_3_N_4_ samples were listed in Table 1. In all cases, not only carbon and nitrogen, which are the main constituent elements of g-C_3_N_4_, but also hydrogen and oxygen were detected. As the synthesis temperature increased, the compositions of carbon and nitrogen became closer to the ideal values, although the carbon content was obviously lower. The hydrogen and oxygen impurities were also reduced with an increase in the synthesis temperature. These results indicate that rising temperature is important to obtain purer g-C_3_N_4_ in terms of the chemical composition.

**Table 1:** Results of elemental analysis and specific surface area measurements.

synthesis temperature [K]	composition [wt %]				specific surface area [m^2^ g^−1^]

	C	N	H	O	

773	32.68	59.60	1.78	4.98	38
823	33.29	59.97	1.53	4.58	36
873	33.64	60.38	1.26	4.15	56
923	34.29	61.14	1.09	2.90	54
ideal C_3_N_4_	39.13	60.87	0	0	–

**Figure 3:**
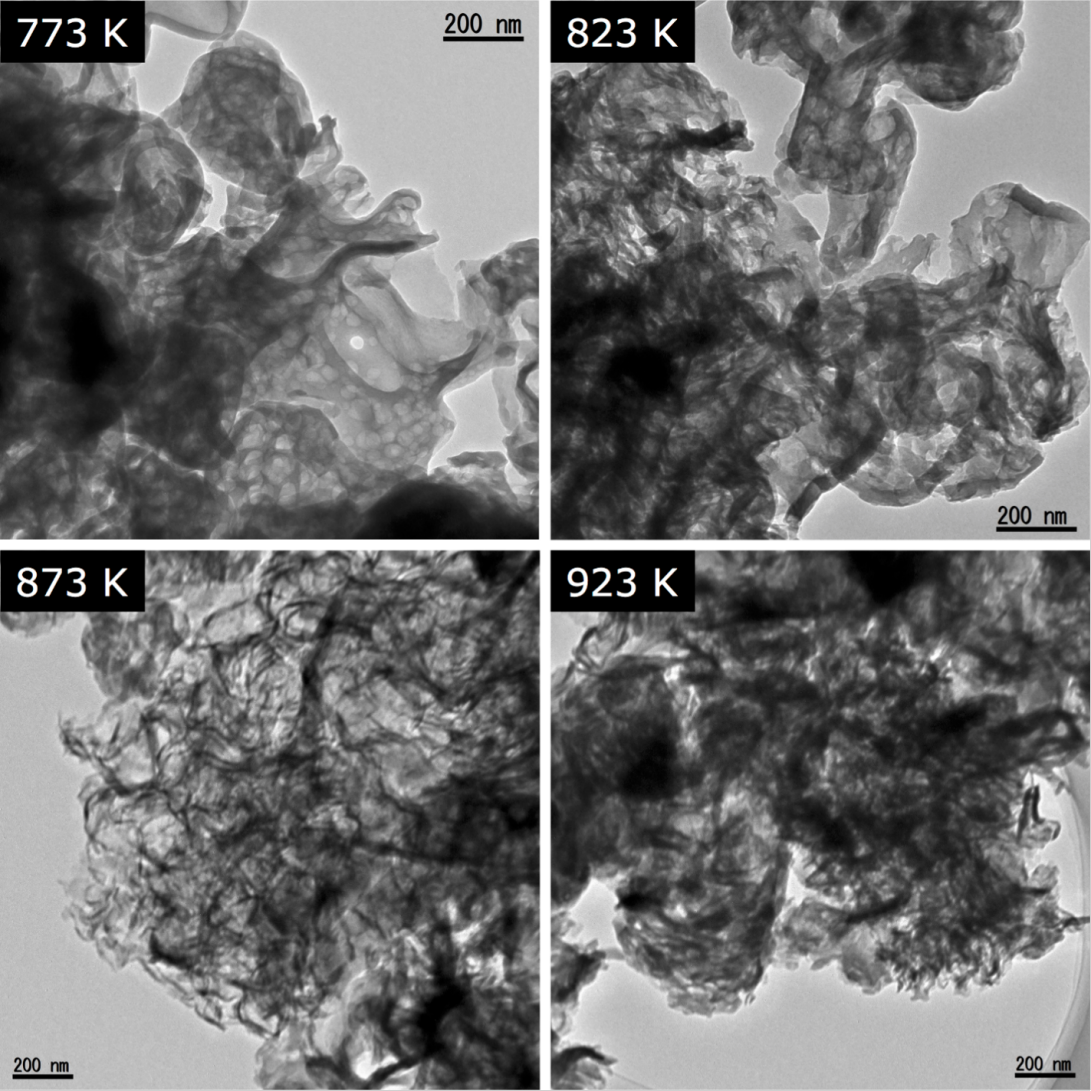
TEM images of g-C_3_N_4_ synthesized at different temperatures.

TEM images of the same samples are shown in Figure 3. The sample synthesized at 773 K had a lot of circular voids having 50–100 nm in size. At 823 K, this void structure was less prominent, and sheet-like morphology started to appear. With a further increase in the synthesis temperature, the synthesized samples consisted of disordered nanosheets. This change in particle morphology is in qualitative agreement with that in the specific surface area (see Table 1).

**Figure 4:**
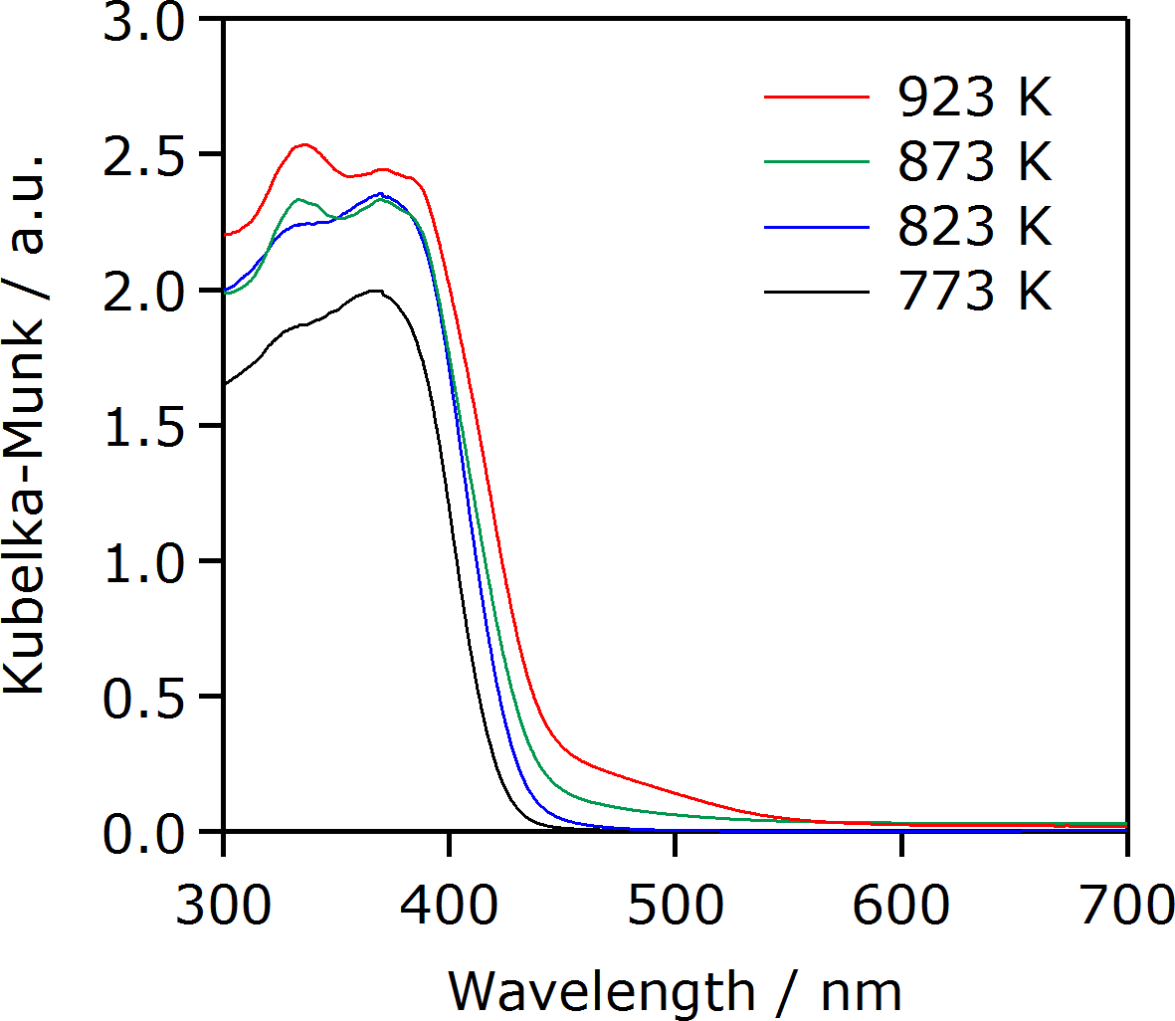
UV–visible diffuse reflectance spectra of g-C_3_N_4_ synthesized at different temperatures.

Figure 4 shows the UV–visible diffuse reflectance spectra of g-C_3_N_4_ synthesized at different temperatures. All of the samples exhibited an absorption edge at 420–450 nm, attributed to electron transitions from the valence band formed by nitrogen 2p orbitals to the conduction band formed by carbon 2p orbitals [25]. The band gaps of the synthesized g-C_3_N_4_ were estimated to be ca. 2.8–3.0 eV, from the onset wavelength of the diffuse reflectance spectra. This value is consistent with that reported previously [24]. As the synthesis temperature increases, the onset wavelength is shifted to longer wavelengths (i.e., band gap is decreased), with more pronounced tailing absorption extending to 550 nm that is assigned to n−π* transitions involving lone pairs on the edge nitrogen atoms of the heptazine rings [28–29]. While the n−π* transitions are forbidden for perfectly symmetric and planar heptazine units, they become partially allowed with increasing the condensation of layers in g-C_3_N_4_, which results from an increase in the synthesis temperature.

### Photocatalytic activities for CO_2_ reduction and H_2_ evolution

**Figure 5:**
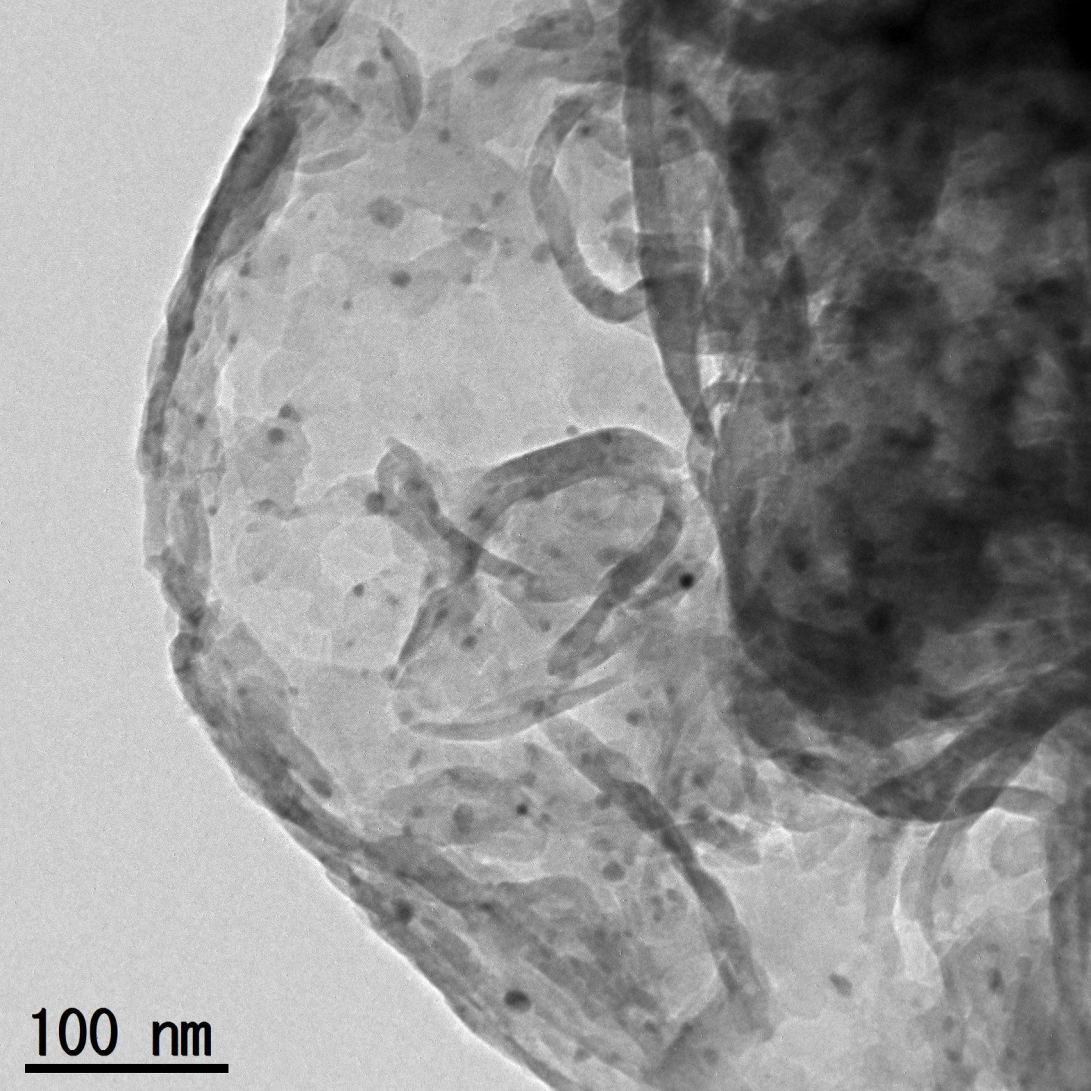
A typical TEM image of Ag-loaded g-C_3_N_4_. The synthesis temperature of g-C_3_N_4_ was 873 K in this case.

Using the as-prepared g-C_3_N_4_, CO_2_ reduction was conducted with the aid of RuP cocatalyst and Ag promoter in a *N,N*-dimethylacetamide (DMA)/TEOA mixed solution under visible light (λ > 400 nm). Here TEOA works as an effective electron donor that scavenges holes generated in the valence band of g-C_3_N_4_ [25]. The use of DMA as the solvent for CO_2_ reduction using RuP/mpg-C_3_N_4_ has previously been shown to be the best choice of solvents to maximize the photocatalytic activity [10]. Because RuP does not absorb visible light efficiently, the g-C_3_N_4_ component can be activated selectively by visible light [10]. Our previous study also indicated that the amount of a molecular cocatalyst is very important in this kind of mononuclear-complex/C_3_N_4_ hybrid photocatalyst for visible-light CO_2_ reduction [8]. To eliminate any other effects other than heating temperature of urea, we fixed the amount of RuP in this study. Ag nanoparticles loaded on mpg-C_3_N_4_ serves as a promoter of electron transfer from mpg-C_3_N_4_ to RuP, as discussed in our previous work [13]. TEM observation indicated that the loaded Ag is in the form of nanoparticles of 5–10 nm in size (Figure 5). Without Ag (i.e., RuP/g-C_3_N_4_), formate production was clearly observable, but the activity was typically 20% that of RuP/Ag/g-C_3_N_4_. Hence we employed Ag as an additional promoter in all cases. It should be also noted that no reaction took place using only g-C_3_N_4_.

As listed in Table 2, the main product during the reaction was formate with 90–95% selectivity. Minor products were CO and H_2_. With increasing the synthesis temperature, the formate generation activity was improved to reach a maximum at 873–923 K, while almost unchanging the CO and H_2_ evolution. The catalytic turnover number of formate generation calculated based on the mole amount of RuP at the optimal conditions reached 650, which confirms the catalytic cycle of the reaction.

**Table 2:** Photocatalytic activities of g-C_3_N_4_ synthesized at different temperatures for CO_2_ reduction and H_2_ evolution under visible light (λ > 400 nm)^a^.

synthesis temperature [K]	CO_2_ reduction^b^ [µmol]			H_2_ evolution^c^ [µmol]

	formate	CO	H_2_	

773	2.8	0.2	0.1	7.0
823	3.0	0.2	0.1	9.7
873	5.2	0.1	0.1	17.9
923	5.1	0.1	0.1	18.6

^a^Reaction conditions: photocatalyst (4.0 mg); reactant solution (4.0 mL); light source, 400 W high-pressure Hg lamp with a NaNO_2_ aqueous solution filter. Reaction time: 5 h. ^b^With 2.0 µmol g^–1^ RuP and 5.0 wt % Ag. Performed in a DMA/TEOA mixed solution (4:1 v/v). ^c^With 3.0 wt % Pt. Performed in a water/TEOA mixed solution (9:1 v/v).

H_2_ evolution was also conducted in a mixed solution of water and TEOA. It is also noted that Pt was in situ loaded on g-C_3_N_4_ as a cocatalyst to facilitate H_2_ evolution. As listed in Table 2, all of the synthesized samples produced H_2_. Similar to the trend in CO_2_ reduction, the H_2_ evolution activity was enhanced as the synthesis temperature was increased.

The results of the photocatalytic reactions thus indicated that photocatalytic activities of g-C_3_N_4_ derived from urea were dependent on the heating temperature of urea. The trend of activity observed in both CO_2_ reduction and H_2_ evolution could be explained in terms of the formation of the g-C_3_N_4_ structure. Physicochemical analyses indicated that increasing the synthesis temperature of g-C_3_N_4_ promotes conversion of urea into g-C_3_N_4_ (Figure 1 and Figure 2), which has even better visible-light absorption (Figure 4). The better light absorption profile as well as higher specific surface area of the g-C_3_N_4_ samples synthesized at elevated temperatures would have a positive impact on the activity to a certain extent. It is also noted that the photocatalytic activity for CO_2_ reduction was much lower than that for H_2_ evolution, even though the same electron donor, TEOA, was employed. This in turn implies that there still remains much room for the improvement of CO_2_ reduction activity, for example, if a proper modification method, which allows for more efficient electron transfer, is developed. This is now under investigation in our laboratory.

## Conclusion

Heating urea in air at 773–923 K resulted in the formation of g-C_3_N_4_, which exhibited photocatalytic activity for CO_2_ reduction into formate under visible light with the aid of a molecular Ru(II) cocatalyst. Experimental results highlighted that higher heating temperature for the synthesis led to the production of more crystallized g-C_3_N_4_ with higher specific surface area and more pronounced visible-light absorption, which was preferable for photocatalytic reactions of both CO_2_ reduction and H_2_ evolution. However, too high synthesis temperature causes a mass loss of g-C_3_N_4_ due to thermal decomposition, which is not practically desirable.

## Experimental

### Materials and reagents

g-C_3_N_4_ samples were synthesized by heating 10 g of urea (99^+^%, Wako Chemicals Co.) in air at a ramp rate of 2.3 K min^−1^ to a given temperature (773–923 K), keeping that temperature for 4 h, then cooling without temperature control.

Ag (5.0 wt %) was loaded as a promoter onto the surface of g-C_3_N_4_ by an impregnation method using AgNO_3_ (>99.8%, Wako Pure Chemicals Co.) as the precursor according to the previous method [13]. g-C_3_N_4_ (50 mg) was dispersed in an aqueous AgNO_3_ solution (10 mL). The water content was subsequently removed under reduced pressure at 317 K. The resulting solid sample was heated under a H_2_ stream (20 mL min^−1^) at 473 K for 1 h.

RuP was synthesized according to methods reported in our previous paper [10]. Adsorption of RuP onto Ag/g-C_3_N_4_ was conducted by dispersing 40 mg of Ag/g-C_3_N_4_ in an acetonitrile (MeCN) solution (20 mL) of RuP. The suspension was stirred overnight at room temperature in the dark to allow for the adsorption/desorption equilibrium, followed by filtration and washing with acetonitrile. The filtrates were collected and concentrated to a volume of 30 mL. The amount of the ruthenium complex absorbed was calculated based on the UV–vis spectrum of the filtrate, using Equation 1:

[1]
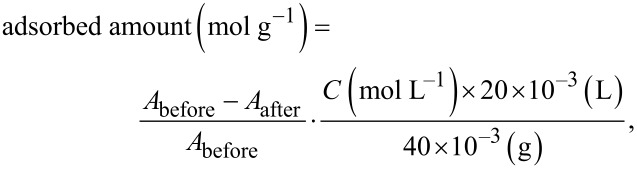


where *A*_before_ and *A*_after_ are the absorbance of the solution before and after the adsorption procedure, respectively, and *C* is the initial concentration of RuP.

Organic solvents used in this work were subject to purification prior to use. DMA was dried over molecular sieves 4 Å (which was heated at 373 K under reduced pressure (<1 Torr) overnight for several days), and distilled under reduced pressure (10–20 Torr). MeCN was distilled over P_2_O_5_ twice, and then distilled over CaH_2_ prior to use. TEOA was distilled under reduced pressure (<1 Torr). The distilled DMA and TEOA were kept under Ar prior to use.

### Characterization

The prepared materials were characterized by X-ray diffraction (XRD) (MiniFlex600, Rigaku; Cu Kα radiation), Fourier-transform infrared (FTIR) spectroscopy (FTIR-610, Jasco), transmission electron microscopy (TEM) (JEM-2010F, JEOL), and UV–visible diffuse reflectance spectroscopy (DRS) (V-565, Jasco). The Brunauer–Emmett–Teller (BET) surface area of each specimen was determined using a BELSOEP-mini instrument (BEL Japan) at liquid nitrogen temperature. The amount of carbon, nitrogen, hydrogen and oxygen were determined by elemental analysis (MICRO CORDER JM10, J-SCIENCE) by Suzukakedai Materials Analysis Division, Technical Department, Tokyo Institute of Technology.

### Photocatalytic reactions

Reactions were performed at room temperature (298 K) using an 8 mL test tube containing 4 mL of solution by dispersing 4 mg of photocatalyst powder. For H_2_ evolution, a mixed solution of water and TEOA (9:1 v/v) was used as the reactant solution, which was in prior purged with Ar for 20–30 min to remove residual air. Pt (3.0 wt %) was loaded in situ using H_2_PtCl_6_ (>98.5%, Wako Pure Chemicals Co.) as the precursor. For CO_2_ reduction, a mixed solution of DMA and TEOA (4:1 v/v) was used. Prior to irradiation, the suspension was purged with CO_2_ (Taiyo Nippon Sanso Co., >99.995%) for 20–30 min. A 400 W high-pressure Hg lamp (SEN) was used as a light source, in combination with a NaNO_2_ solution as a filter to provide visible light irradiation (λ > 400 nm). The gaseous reaction products were analyzed using a gas chromatograph with a thermal conductivity detector (GL Science, Model GC323). The formate generated in the liquid phase was analyzed via a capillary electrophoresis system (Otsuka Electronics Co., Model CAPI-3300).
